# Endocrine profiling of reproductive status and evidence of pseudopregnancy in the Pacific walrus (*Odobenus rosmarus divergens*)

**DOI:** 10.1371/journal.pone.0239218

**Published:** 2020-09-15

**Authors:** Jenell T. Larsen Tempel, Shannon Atkinson

**Affiliations:** Fisheries Department, College of Fisheries and Ocean Sciences, University of Alaska Fairbanks, Juneau Campus, Juneau, Alaska, United States of America; University of Missouri Columbia, UNITED STATES

## Abstract

Endocrine profiling is an increasingly utilized tool for detecting pregnancies in wild populations of mammals. Given the difficulty in calculating reproductive rates of Pacific walruses (*Odobenus rosmarus divergens*) the use of endocrine techniques for determining pregnancy rates could be particularly useful for management of the population. The goals of this study were to 1) determine if progesterone and total estrogen concentrations in ovarian tissues of female walruses could be used to determine reproductive state and 2) determine if walruses undergo a functional postpartum estrus, as is seen in other pinnipeds. Ovaries were collected from female walruses (*n* = 13) hunted in subsistence hunts by Alaska Native communities. Females were categorized as postpartum, full-term pregnant, pregnant diapause or unbred. Total estrogen concentrations were greatest in unbred (*n* = 2) and pregnant (*n* = 2) females. Progesterone concentrations were also nominally larger in unbred (*n* = 2) than pregnant (*n* = 2) and postpartum (*n* = 9) animals. Small samples sizes precluded the use of statistical comparisons among groups. Corpora lutea tissue samples in this study did not reflect the presence of a postpartum estrus in the month of May as postpartum females yielded lower total estrogen concentrations than unbred or pregnant animals. Both unbred animals were in a state of pseudopregnancy, which has not been physiologically described for this species before. The progesterone profiles in late (59 ng/g) and early (140 ng/g) pregnancy were lower than expected and fell within the range of the postpartum females (36–210 ng/g), suggesting low production of the hormone by the corpus luteum during these phases of pregnancy. Profiling reproductive hormones in free-ranging walruses demonstrates that an endocrine approach may be a valuable tool for determining reproductive status of females, however increased sample sizes and time of year must be considered to accurately separate pregnant versus pseudopregnant individuals.

## Introduction

Reproductive hormone profiles can be used to detect pregnant and non-pregnant animals, and has been demonstrated in multiple marine mammal species [1–3]. For this reason, endocrinology has gained traction as a tool for determining pregnancy in animals in human care, as well as in wild populations. Most of the research on female reproductive hormones in wild populations has focused on cetacean species, including common bottlenose (*Tursiops truncatus*) [4], long-beaked (*Delphinus capensis*) [5], short-beaked common (*Delphinus delphis*), Northern right whale (*Lissodelphis borealis*), Pacific white-sided dolphins (*Lagenorhynchus obliquidens*) [6], and pilot (*Globicephala melas*) [4], minke (*Balaenoptera acutorostrata*) [7, 8], fin (*Balaenoptera physalus*) [9], blue (*Balaenoptera musculus*) [10, 11], humpback (*Megaptera novaeangliae*) [12] and bowhead whales (*Balaena mysticetus*) [13]. Fewer studies exist for pinnipeds and many of them have been carried out on animals in human care, which may not follow the life history timing or seasonal reproductive patterns witnessed in conspecifics in their native environments [14–17]. Studies on progesterone and estrogen concentrations in pinnipeds have shown that these endocrine markers are able to detect pregnancy and sometimes estrus in Pacific walruses (*Odobenus rosmarus divergens*) [18], Hawaiian monk seals (*Monachus schauinslandi*) [19], harbor seals (*Phoca vitulina)* [20], and Steller (*Eumetopias jubatus*) [21], California (*Zalophus californianus*) [22], and Galapagos sea lions (*Zalophus wollebaeki*) [23]. In addition to reproductive hormones, lipids play an important role in reproduction. Specifically, carbohydrates and fatty acids have been demonstrated to be important energy sources for oocyte and embryo development in mammals [24–27]. Studies on marine mammals have been limited to analyzing lipid content in blubber to determine reproductive status [28]. In the present study, percent lipids of ovarian tissues were investigated to determine if they were a useful indicator of reproductive status.

**Fig 1:**
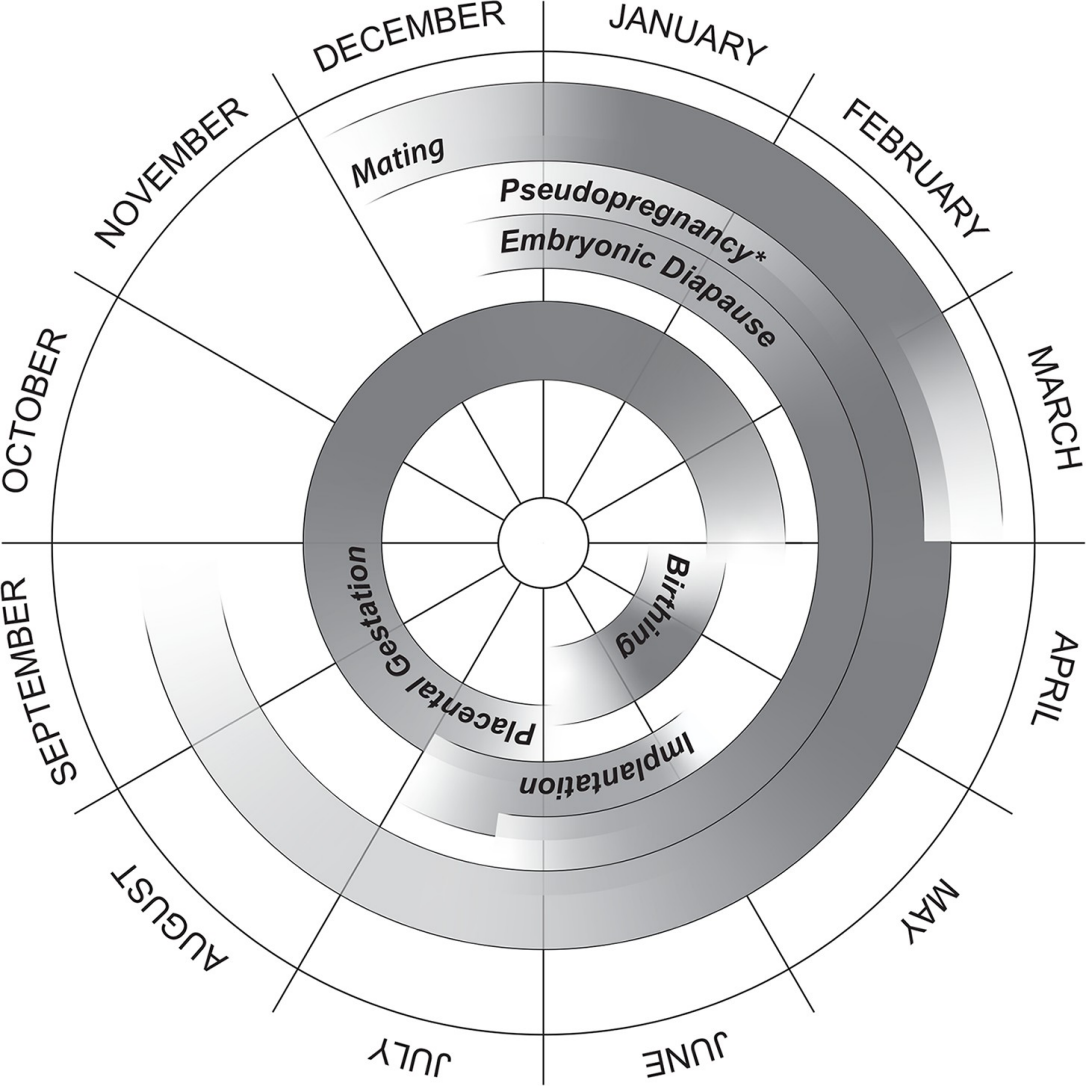
The reproductive cycle of the female Pacific walrus (adapted from Fay 1982). Findings from the present study indicate that some females may experience a pseudopregnancy if they do not successfully breed (indicated by an asterisk).

Endocrine profiles of reproductive hormones are useful in determining reproductive status because they reflect ovarian activity. In female mammals two main hormones govern the reproductive cycle: estrogens and progesterone. There are three primary types of estrogen: estrone, estradiol-17β, and estriol [29]. Estradiol is the principle product produced during the follicular phase, however estrone sulfate or “total” estrogens are often easier to measure [19, 30]. Estradiol is the estrogen associated with ovulation and it is responsible for cellular proliferation and hydration. Both progesterone and estrogens are produced in the ovaries. Estradiol is produced in the thecal and granulosa cells within the ovarian follicles as they enlarge in preparation for ovulation [30]. Estradiol levels spike, prior to ovulation and then quickly decline. Following ovulation, the newly formed corpus hemorrhagicum develops into the corpus luteum (CL) and begins producing progesterone whether or not the animal is pregnant. The role of progesterone is to prepare the uterus and sustain pregnancy, if conception occurs. If the female is not pregnant, the CL will regress, and progesterone production will decrease. However, if pregnancy occurs, progesterone will remain high throughout gestation [31, 32]. Yet, some species exhibit high levels of progesterone as well as behavioral indicators of pregnancy, when they are not pregnant [33]. This “false pregnancy” has been termed pseudopregnancy and occurs primarily in copulation-induced ovulators [33]. Few published studies have profiled reproductive hormones in the tissues of free-ranging walruses [18]. The Pacific walrus has a unique reproductive cycle among pinnipeds, most of which have an annual breeding cycle which includes a postpartum estrus in spring or summer followed by an embryonic diapause [30]. In brief, the reproductive cycle of female walruses is characterized by mating during December-March, embryonic diapause until June-July at which point nidation occurs, and a 15-16-month total gestation period, during which 8–11 months are active gestation [34] (Fig 1). There have been several reports of calves being born outside of this “established” window, during winter months [34]. It is unclear if these were premature births or if these were rare instances in which females were breeding beyond December-March. If these females were following a 15–16 month gestation, it would require breeding in May-July, coinciding with parturition. Based on anatomical studies finding CL within the ovaries of females as late as August, Fay [34] determined the species to have a postpartum estrus. However, he recognized that this postpartum estrus was likely outside the fertility window of breeding males, therefore this diestrus cycle could be considered functionally a monoestrus cycle. Similarly, in Atlantic walruses (*Odobenus rosmarus rosmarus*), Born [35] found evidence of ‘extra-seasonal’ ovulations (by presence of CL outside of the rut) which he characterized as pseudopregnancies and attributed to young females approaching sexual maturity. However, these determinations were made solely using an anatomical approach from harvested ovaries. Using an endocrinological approach, we investigated for the first time whether females indicate physiological signs of estrus, via elevated estrogen concentrations following parturition.

In the present study, reproductive tracts were opportunistically sampled from Pacific walruses hunted by Alaska Native hunters, to determine if endocrine profiles in ovarian tissues were indicative of the known reproductive status of the harvested female. The objectives were to 1) examine whether females exhibit endocrine indicators of a postpartum estrus or functional diestrus, 2) profile progesterone and total estrogen concentrations and percent lipids in CL of female Pacific walruses in four known reproductive states, and 3) determine whether follicular activity is a good indicator of ovarian activity by analyzing the number, size and endocrine profiles of follicles. This study adds to our knowledge of walrus reproductive ecology by ascertaining whether endocrine profiles are useful indicators for determining reproductive status of adult female walruses. In addition, the use of hunted specimens allows for anatomical confirmation of the endocrine measurements.

## Materials and methods

### Ethics statement

All ovarian tissues were obtained from female walruses harvested by Alaska Native subsistence hunters in the communities of Gambell and Savoonga on St. Lawrence Island, Alaska, during May of 2011, 2015 and 2016. All samples were collected by US Fish and Wildlife Service (USFWS) in accordance with Federal regulations 50 CFR 18.23(b)(2) per the Walrus Harvest Monitoring Program.

### Sample collection

A total of 13 sexually mature females were sampled. Females were harvested in the month of May during which they could be categorized as one of four different reproductive states: postpartum (*n* = 9), full-term pregnant (*n* = 1), embryonic diapause (*n* = 1) and unbred (*n* = 2) (Fig 1, Table 1). Reproductive states were determined using information provided by hunters about each animal harvested and by gross dissection of the ovaries and reproductive tracts when available. Hunters were trained by USFWS personnel and given field notebooks to document each animal hunted. Sexual maturity was determined by presence of a CL within the ovary [36–39]. Ovaries indicated if the female was reproductively active during her current cycle by presence of the CL, whereas ovaries lacking a CL were termed inactive. The presence of an active CL two or more months post-mating season, without the presence of a fluid filled amnion or other early pregnancy-related indicators in the uterus, such as a blastocyst implanted within the uterine horn, was used to define a physiological pseudopregnancy. Hunter observations indicated if a newborn calf and/or yearling was present with the adult and if she was lactating. Females that were not pregnant, nor with a newborn calf, were considered unbred. We did not consider lactation to be indicative of a recent breeding as females may nurse calves for two years and sometimes longer [34], therefore lactating females could be categorized as unbred animals if they did not have a newborn calf or show signs of a current pregnancy. When only ovaries were available for analysis, a female documented with a newborn calf and lactating was considered postpartum; when a reproductive tract was present a fresh zonal scar from placentation confirmed hunters’ observations. All hunter observations of females with newborn calves were confirmed when reproductive tracts were present (*n* = 7), providing confidence in using hunter knowledge of reproductive status when full reproductive tracts were not available (*n* = 2) (Table 1). One female was categorized as full-term pregnant based on the hunter observation of a fully developed fetus in the uterine horn of the female, and that the timing of the hunt on St. Lawrence Island coincides with peak calving for Pacific walruses [34]. The uterus of the full-term pregnant animal was not collected. One reproductive tract was collected that contained an amniotic sac indicative of a female in embryonic diapause. Any unbred female with a CL during the sampling period (in May) was considered to be exhibiting diestrus as the breeding window generally occurs December-March.

**Table 1:** Life history data on female Pacific walruses used in the present study.

Animal ID	Age (years)	Lactating	Harvest date	Offspring present	Tract present	Reproductive status	Progesterone (ng/g)	Total estrogens (ng/g)	
							CL	CL	OT
G160013	13	Y	5/13/2016	calf and yearling	Y	postpartum	63.2	16.0	n/a
G160048	n/a	Y	5/14/2016	calf	N	postpartum	59.3	10.6	11.1
S110004	7	Y	5/21/2011	calf	Y	postpartum	33.0	9.9	4.6
G110035	10	Y	5/8/2011	calf	Y	postpartum	40.7	14.9	1.9
S110021	11	Y	5/16/2011	calf	Y	postpartum	40.2	14.7	n/a
G110286	16	unknown	unknown	unknown	Y	postpartum	167.3	11.8	4.0
S110002	16	Y	5/16/2011	calf	Y	postpartum	36.1	5.7	n/a
G110044	20	Y	5/8/2011	calf	Y	postpartum	47.7	34.1	3.3
S110008	n/a	Y	5/20/2011	calf	Y	postpartum	209.8	n/a	n/a
G160053	n/a	Y	5/13/2016	fetus	N	full-term pregnant	58.5	193.7	5.1
G160045	13	unknown	unknown	unknown	Y	pregnant diapause	140.3	205.4	16.4
G150005	19	Y	5/6/2015	yearling	Y	unbred	1533.0	368.9	43.8
G110290	16	unknown	unknown	unknown	Y	unbred	2007.2	1368.8	5.3

Hormone profiles were conducted on corpora lutea (CL) and ovarian tissues (OT), age is shown in years, and animal ID indicates whether the female was hunted from the community of Gambell (G) or Savoonga (S), the date hunted, and the animal number.

Samples were not unbiased as hunters on St. Lawrence Island typically select for female-calf pairs. This skewed our samples toward sexually mature, breeding females, which was appropriate for our study as we sought to compare the endocrine profiles of pregnant and non-pregnant adult females. Morphometrics were taken including: tract weight, placental zone width, uterine horn width, and CL weight and dimensions. Tracts were stored frozen and allowed to thaw before analysis. Excess fluid was absorbed from the tracts and they were weighed on a hanging scale to the nearest 0.5 lbs and converted to kg. The width of the zonary placental scar was measured inside the dissected uterine horn, to the nearest 0.5 cm. Uterine horn width was measured from the proximal edge of the broad ligament directly outward to the greatest width of each uterine horn; widths were recorded to the nearest 0.5 cm. Ovaries were removed from the reproductive tract. Ovaries were examined for the presence of CL and follicles. CL were identified by their size (taking up to ^1^/_3_ to ^1^/_2_ of the ovarian size), spherical shape, firm texture and often yellowish color [34]. The CL were removed, trimmed of adjacent ovarian tissues and weighed to the nearest 0.05 g. The interior section of the CL was used for hormone analyses ensuring that no surrounding ovarian tissues were included. Follicles were located on the serosal surface of the ovary and ranged from <1 mm to 5 mm. Antral follicles were spherical shape and the antrum was filled with a translucent fluid. The number of follicles per ovary were counted, excluding those ≤1 mm, as at that size they are no longer grossly distinguishable. Follicles were measured with calipers to the nearest millimeter. The following size classes were used: small (≤1 mm), medium (2–4 mm) and large (>4 mm). Those that were of medium or large size were aspirated with a syringe and analyzed for total estrogen concentrations; follicles ≤1 mm were unable to be aspirated and were therefore excluded from hormone analysis. For this reason, more than one follicle per female was analyzed as some animals yielded multiple medium and large-sized follicles, while others contained only small follicles. In addition to follicular fluid, ovarian tissue samples were analyzed separately for hormone concentrations and excluded follicles in the medium size class or larger. Ovarian tissues were sampled from both active and inactive ovaries (i.e., those that had a CL or not, respectively).

### Tissue extraction

The extraction process for both the CL and ovarian tissues follows the methods of Mansour et al. and Kellar et al. [6, 7] as modified by Atkinson et al. [10]. Briefly, a section of CL/ovarian tissue for each female was cut with a scalpel and manually homogenized with a Teflon-tipped hand tool in a sterile 12 x 75 mm borosilicate glass test tube. One ml of ethanol was added and the tissue was homogenized for 3–5 minutes per sample in the ethanol. Samples were centrifuged at 3000 rcf for 15 minutes. The ethanol was transferred to new test tubes and an additional 1 ml of ethanol was added to the tissues and samples were again centrifuged at 3000 rcf for 15 minutes. The resulting supernatant was 2 ml. Supernatants were evaporated under compressed air overnight. A 4:1 solution of ethanol to acetone was created and 1 ml of the solution was added to each sample and vortexed for one minute. Samples were centrifuged for 15 min at 3000 rcf. Supernatants were transferred to new test tubes and dried under compressed air. One ml of ether was added to dried samples and samples were vortexed for 10 seconds. Samples were centrifuged (3000 rcf for 15 minutes). Supernatants were again transferred to new test tubes. One ml of acetonitrile was added to each sample; samples were vortexed (10 seconds). Then 1 ml of hexane was added to each sample and they were vortexed again (10 seconds). The acetonitrile layer was collected. This step was repeated and the acetonitrile collected was approximately 2 ml. Samples were dried under compressed air.

Extractions were not necessary for follicular fluid. The follicular fluid was therefore added to the assay buffer in the volume appropriate for the dilution based on the limited amount of fluid, ranging from 2–10 μl. Because follicular fluid is present in ovarian tissues, the validation of ovarian tissues was assumed to be sufficient as validation for follicular fluid.

### Total estrogens radioimmunoassays

A double antibody radioimmunoassay (RIA) kit (MP Biomedicals, Solon, OH) was validated for total estrogens in CL and ovarian tissues of walruses. A pool of CL tissue, including females of all reproductive states, was used in the validation. This was done for two reasons: 1) sample sizes were limited and 2) to ensure proper dilutions could be achieved for females in varying reproductive states. For ovarian tissues, females in all states, except the full-term pregnant state, were used due to small sample sizes. To assess assay parallelism, pooled tissues were serially diluted in assay buffer and assayed in comparison with known doses of total estrogen standards. Slopes were compared for parallelism of the linear portion of the curve, in which samples were analyzed. Serial dilutions of the pooled CL (1:1000 to 1:4000) and ovarian tissues (neat to 1:80) yielded displacement parallel to that of the total estrogen standard curve. Samples were run in duplicate following manufacturer instructions with the exception that volumes were halved and an additional standard (one-half of the lowest standard) was added to the curve to increase sensitivity [22, 23, 40].

Assay accuracy was tested by spiking standard curves with the pooled tissues in equal volumes and were assayed in comparison with the standards spiked only with the assay buffer. Pooled tissues were spiked with 7 total estrogen standards (1.25, 2.5, 5, 10, 25, 50 and 100 pg/ml) for both the CL and ovarian tissues. The measured values of the total estrogens (with the total estrogens measured in un-spiked samples subtracted) were compared against the expected values of the standards. Inter-assay coefficient of variation for low, medium and high controls for all assays were 4.4%, 13.6% and 12.9%, respectively, and intra-assay coefficients of variation for all assays were <10%. Samples with an intra-assay coefficient of variation >10% were re-diluted as appropriate and re-assayed. Mean sensitivity was 0.3 ng/g ± 0.04 SE (*n* = 5). Total estrogen concentrations were profiled from CL (*n* = 12), ovarian tissues (*n* = 9) and follicles (*n* = 12). Table 1 provides sample sizes for each tissue relative to reproductive status. All CL samples had a mass of ≥100 mg except in one case; ovarian tissue samples ranged from 50–200 mg.

### Progesterone enzyme immunoassays

Progesterone concentration was determined via enzyme immunoassay (EIA) kits purchased from Enzo Life Sciences (ADI-900-011). Standard concentrations were determined according to kit instructions and samples were run in duplicate. As with the total estrogens, a pool of CL tissue including females of all reproductive states was used in the validation for progesterone. The same steps were used for determining assay parallelism and accuracy as with total estrogens. When assessing parallelism, serial dilutions of the pooled tissues (1:200 to 1:3200) yielded displacement parallel to that of the total progesterone standard curve.

To determine assay accuracy, pooled CL tissues were spiked with 6 progesterone standards (15.62, 31.25, 62.5, 125, 250, and 500 pg/ml). Inter-assay coefficient of variation for low, medium and high controls were 17.5%, 7.7% and 11.2% respectively and intra-assay coefficient of variation was <10%. Samples with an intra-assay coefficient of variation >10% were re-diluted as appropriate and re-assayed. Mean sensitivity was 6.58 ng/g ± 0.23 SE (*n* = 5). Progesterone concentrations were profiled from CL (*n* = 13). All samples had a mass of ≥ 200 mg.

### Lipid analysis

A modified Folch method was used to measure percent lipids [41]. For CL tissues samples sizes were as follows: postpartum (*n* = 9), unbred (*n* = 2), full-term pregnant (*n* = 1), and embryonic diapause (*n* = 1); for ovarian tissues: postpartum (*n* = 5), unbred (*n* = 1), full-term pregnant (*n* = 1) and embryonic diapause (*n* = 1). Tissue samples weighed 5–40 mg. Each sample was combined with 5 ml of hydromatrix and compacted into an Accelerated Solvent Extractor (ASE) cell (Thermo Dionex ASE 350). The remaining volume in each ASE cell was filled with sand and cells were loaded into the ASE machine. A two-part chloroform: one-part methanol solvent was created and all samples were rinsed twice with the solvent. Afterwards, the volume of the samples was calculated and 0.88% potassium chloride was used to separate the organic fraction. Potassium chloride was added to each sample in a volume that was one-fourth of the sample’s volume after the rinse. The aqueous layer was discarded and the organic layer was collected carefully so that no amount of aqueous solution contaminated the sample. A Rotovap (Heidolph Laborota 4011/HB Digital) was used to concentrate the sample to just under 1 ml using a rotation speed of 165–200 rpms. Samples were pipetted into labeled tin pans and dried overnight in a vacuum oven. Percent lipids were calculated using the dried weights of the samples, using the equation:

$$\%Lipids=\frac{\left(X_{1}-X_{2}\right)}{X_{3}}\times 100$$

Where X_1_ is the weight of the organic fraction plus the pan weight, X_2_ is the weight of the empty pan, and X_3_ is the weight of the original tissue.

### Data analysis

**Fig 2:**
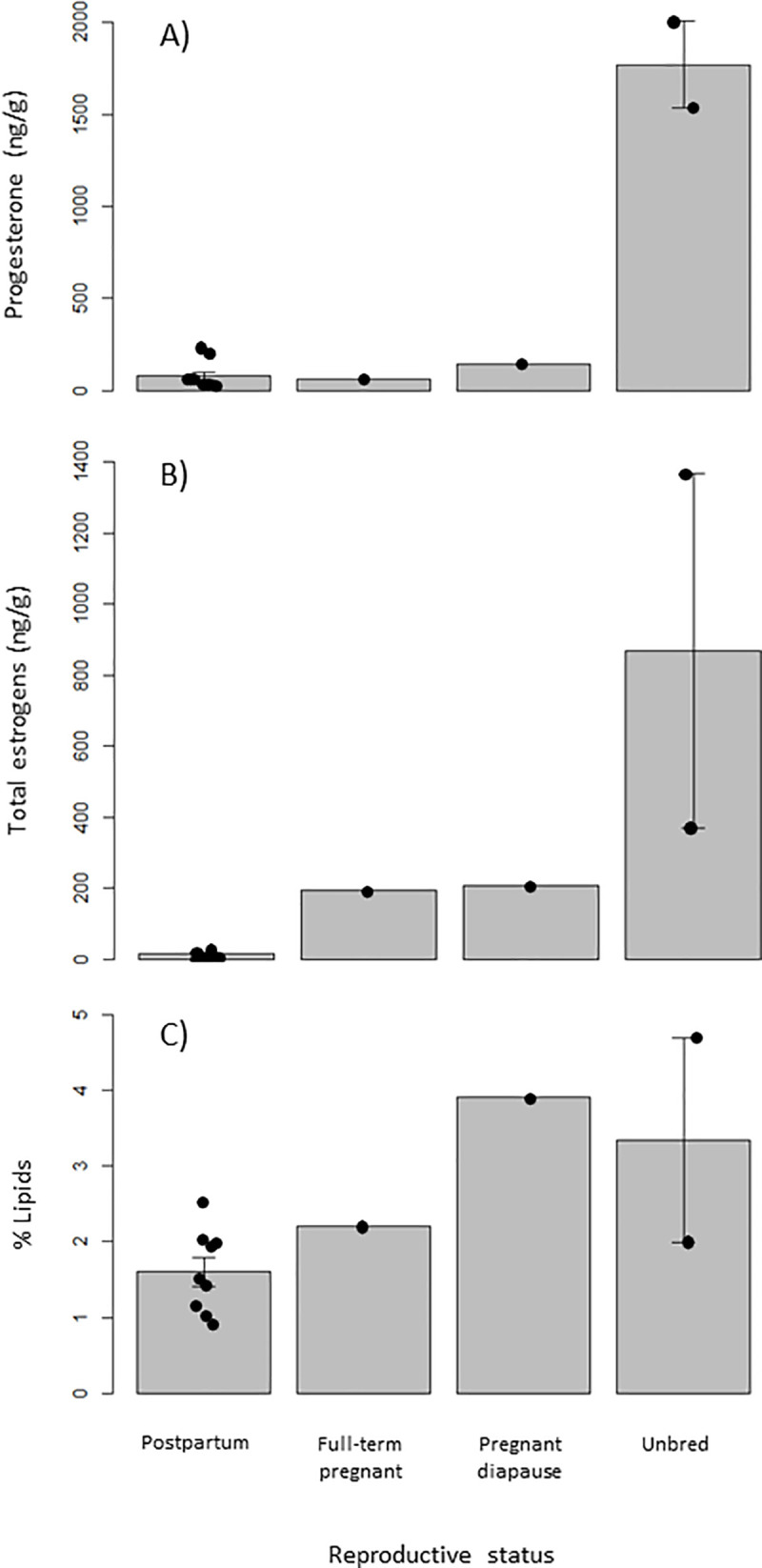
Mean concentrations of (A) progesterone (ng/g), (B) total estrogens (ng/g), and (C) percent lipids in CL of female Pacific walruses in four reproductive states: postpartum, full-term pregnant, pregnant diapause, and unbred. Animals were hunted by Alaska Native hunters near St. Lawrence Island during May 2011, 2015 and 2016. Error bars represent standard errors of the means.

**Fig 3:**
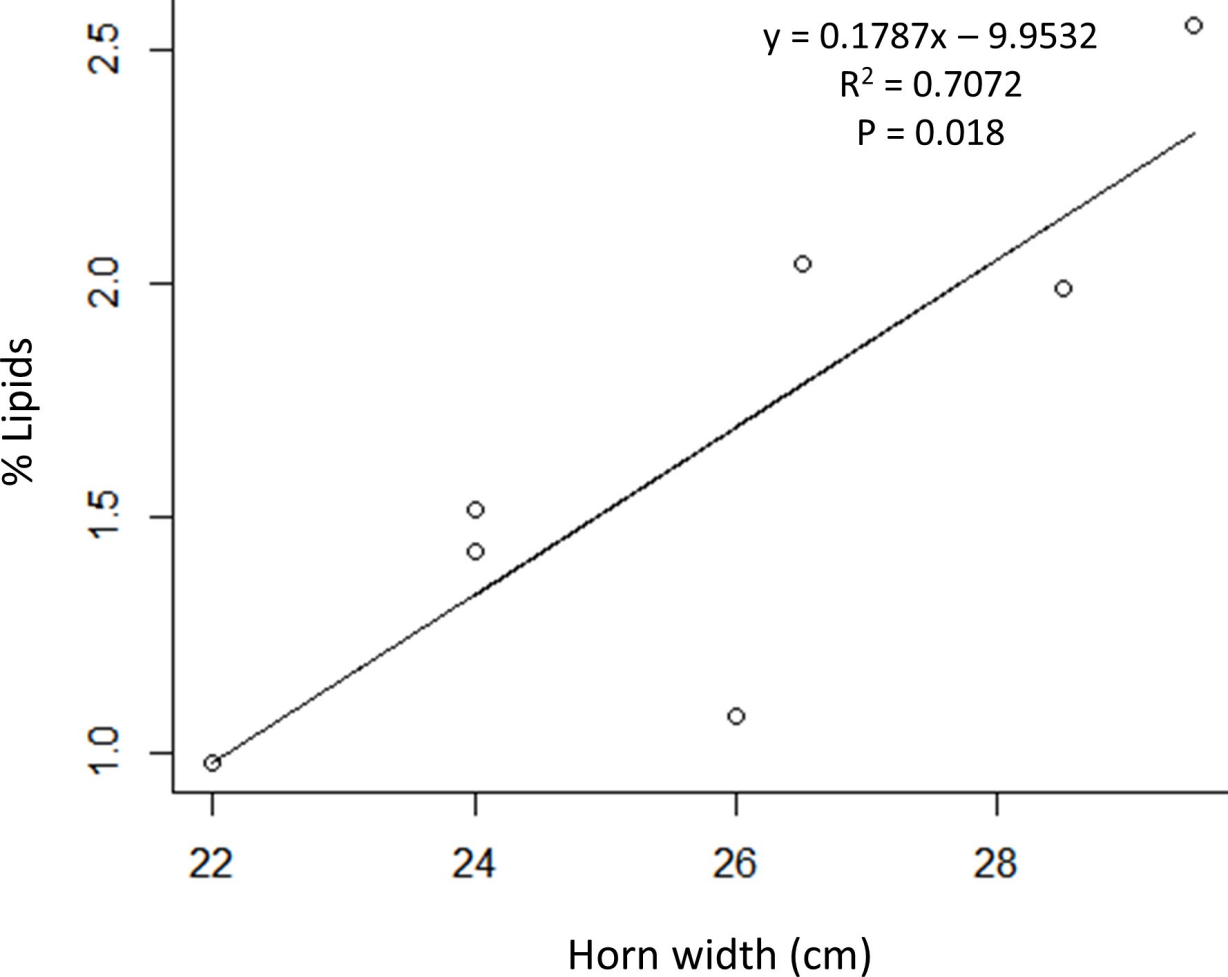
The positive, linear relationship between percent lipids in corpora lutea tissues and uterine horn width in postpartum female Pacific walruses hunted by Alaska Native hunters near St. Lawrence Island, Alaska, in May 2011.

**Fig 4:**
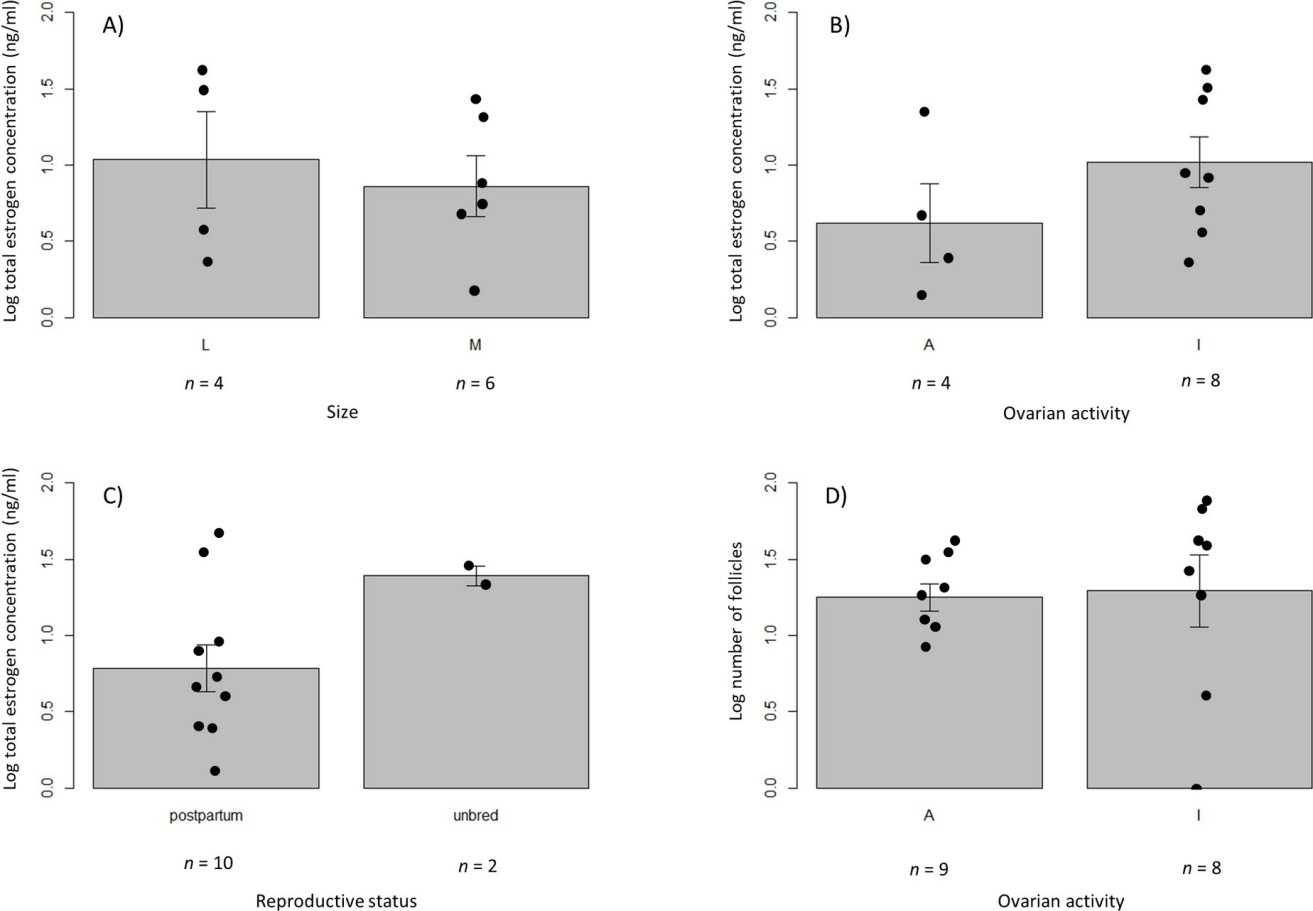
Follicular activity in Pacific walruses hunted by Alaska Native hunters near St. Lawrence Island during May 2011, 2015 and 2016. (A) Total estrogen concentrations (ng/ml) in follicles for large (L) and medium (M) follicles, (B) total estrogen concentrations (ng/ml) in follicles in active (A) versus inactive (I) ovaries, (C) total estrogen concentrations (ng/ml) in follicles of postpartum and unbred females, and (D) the number of follicles in active versus inactive ovaries. All values are presented in a log scale (log_10_). Error bars represent standard errors of the log transformed means.

Assay parallelism was assessed using Student’s *t*-tests [42] to compare the slopes of the linear portion of the curves for the standard curves versus the serially diluted pools. Student’s *t*-tests were conducted in Excel. Mean estrogen profiles within the follicles of large versus medium follicle sizes and for active and inactive ovaries, as well as the number of follicles per active and inactive ovary were compared using t-tests. Simple linear regressions were used to determine relationships between percent lipids in ovarian tissues and harvest dates, animal ages, CL weights, CL dimensions, reproductive tract weights and placental zone width of the uterine horn. RStudio, version 3.6.1 [43] was used for comparing means, linear regressions and creating Figs 2–4. Unfortunately, small samples sizes for hormone analyses did not allow for statistical comparisons among groups. Rather, trends are presented graphically and qualitative differences and similarities in hormone concentrations are described for each of the four reproductive states. Data were log transformed using log_10_ to meet normality assumptions of parametric tests. However, when statistics were not conducted due to small sample sizes, figures show the true values.

## Results

### Assay validations

Both the RIA and EIA demonstrated acceptable parallelism and accuracy validations for the CL and ovarian tissues. For parallelism tests, the slopes of the serially diluted pools did not show statistically significant differences from slopes of the standard curve (for the total estrogens CL pool t = 1.217, P = 0.348 and ovarian tissues pool t = 0.393, P = 0.732; for the progesterone CL pool, t = 0.134, P = 0.906;). For accuracy tests, the results of the observed dose were plotted against the known standard dose and assessed for linearity, slope and y-intercept, displayed as a regression equation; closeness of fit between the y-intercept and observed dose of the pool is shown as the r^2^ value [44]. Recovery of added total estrogens from the CL pooled tissues was 87.04% (y = 0.725x + 0.1698, r^2^ = 0.983). Recovery of added total estrogens from the pool of ovarian tissues was 127.50% (y = 0.840x + 4.778, r^2^ = 0.817); this increased value likely reflects the presence of elevated estrogens in the ovarian tissues used to conduct the accuracy test. Recovery of added progesterone from pooled tissues was 72.04% (y = 0.827x + 0.363, r^2^ = 0.9101). For accuracy tests, slopes of the observed vs. expected concentrations were between 0.7–1.30 [36] and r^2^ values were 0.817–0.983.

### Corpora lutea

Total estrogen profiles were 1–2 orders of magnitude lower in postpartum females (mean = 14.7 ± 3.0 ng/g; *n* = 8) than females in the other three reproductive states (Fig 2). Mean concentrations for total estrogens for unbred females were 868.9 ng/g (368.9–1368.8 ng/g) (*n* = 2), for the full-term pregnant female was 193.7 ng/g (*n* = 1), and for the female in embryonic diapause 205.4 ng/g (*n* = 1). As with the total estrogen concentrations, the unbred females had the greatest concentrations of progesterone in their CL tissues, 1770.1 ng/g (1533.0–2007.2 ng/g) (*n* = 2), whereas the full-term pregnant female was 58.5 ng/g (*n* = 1), the female in embryonic diapause was 140.3 ng/g (*n* = 1), and the postpartum females had a mean of 77.5 ± 64.7 ng/g (*n* = 9). In both hormone profiles, the values for the unbred females did not fall within the range of postpartum (progesterone 33.0–209.8 ng/g and total estrogens 5.7–34.1 ng/g) or pregnant females (full-term pregnant: progesterone 58.5 ng/g and estrogen 193.7 ng/g; pregnant diapause: progesterone 104.3 ng/g and estrogen 205.4 ng/g) but were nominally larger, providing evidence of a physiological pseudopregnancy in the unbred females in our sample (Fig 2). Percent lipids of CL appeared similar across all reproductive states, however mean values were approximately double for both pregnant 3.06% (2.20–3.91%) (*n* = 2) and unbred females 3.34% (1.99–4.69%) (*n* = 2) than postpartum females 1.60 ± 0.58% (*n* = 9).

Nine samples contained both the ovaries and full reproductive tracts (S110004, G110035, S110021, G110286, S110002, G110044, S110008, G160045, and G110290), of these, seven were postpartum females (Table 1) yielding placental scaring in the zone of attachment. There was a significant, positive correlation with percent lipids in the CL and the horn width in the pregnant horn of the postpartum females (y = 0.1787x – 9.9532, r^2^ = 0.7072, P = 0.018) (Fig 3). For these females, the percent lipids in the CL did not have any correlation with the harvest date, animal age, CL weight, CL dimension, reproductive tract weight or placental zone width (P > 0.05).

### Ovarian tissues

Mean estrogen concentrations for ovarian tissues from postpartum females were 5.0 ± 3.6 ng/g (*n* = 5), from unbred females 24.5 ng/g (5.3–43.8 ng/g) (*n* = 2), from the full-term pregnant female 5.1 ng/g (*n* = 1), and from the female in embryonic diapause 16.4 ng/g (*n* = 1). Only one unbred female (G150005) tissue sample was available for lipid analysis. Percent lipids were variable in postpartum females, and ranged from 0.78–2.32%. Percent lipids were as follows for postpartum, full-term pregnant, embryonic diapause and unbred females respectively: 1.60 ± 0.71% (*n* = 5), 0.66% (*n* = 1), 1.24% (*n* = 1) and 2.66% (*n* = 1).

### Follicular activity

Estrogen concentrations in follicular fluid were approximately double in unbred (*n* = 2) females than postpartum females (*n* = 10) (Fig 4C) (S1 Table). The mean concentration for unbred females was 24.9 ng/ml (21.2–28.5 ng/ml), whereas for postpartum females was 11.2 ± 14.4 ng/ml. Trends in the data suggested that differences did not exist between estrogens and follicle size (*n* = 10, P = 0.670) (Fig 4A) (S1 Table), nor estrogen concentrations in follicles in active versus inactive ovaries (*n* = 12, P = 0.246) (Fig 4B) (S1 Table), nor in the number of follicles in active versus inactive ovaries (*n* = 17, P = 0.875) (Fig 4D) (S2 Table).

## Discussion

Profiling hormone concentrations in the tissues of walruses demonstrates that endocrine analyses are potentially useful indicators in determining reproductive states of this species. While this has been done in multiple species with many types of matrices [2, 3], no studies using ovarian tissue of marine mammals for endocrine analyses were found in published literature. The ovarian tissues used in this study are unique in that they are the source location in which progesterone and estrogens are produced, rather than the more commonly sampled matrices used in endocrinology studies of marine mammals such as blood, blubber, and feces [2, 3]. This allowed the measurement of sex steroid hormones from the animal at the time of sampling and directly from the production site. Studies on cetaceans have found the rate at which progesterone is transferred from blood serum to blubber is variable by species. In bowhead whales, progesterone may take weeks to months before concentrations in blubber reflect that of blood [13], however, in bottlenose dolphins progesterone concentrations in blubber may reflect blood serum concentrations just days prior to sampling [45]. Ultimately, using CL tissues allowed for the assessment of females that may be in estrus, which due to the rapid nature of the preovulatory estrogen surge, might be missed when sampling other matrices. Thus, this study adds new information to the unique and understudied reproductive cycle of free-ranging walruses.

### Total estrogens

A rapid increase in total estrogens is preovulatory in mammals and results in ovulation and the subsequent formation of the CL. Because both of the unbred animals exhibited a CL and none of the postpartum females exhibited morphological signs of estrus (follicles ≥ 19 mm), none of the sampled females were considered to be exhibiting a postpartum estrus. The total estrogen concentrations in CL of pregnant and unbred females were 1–2 orders of magnitude greater than that of postpartum females (Fig 2B). Total estrogens in CL were 59 times greater in unbred than postpartum animals. One unbred female (G110290) yielded 1368.8 ng/g total estrogens, whereas the mean total estrogen concentration for postpartum females was 14.7 ng/g. Unbred female G110290 did not have a dominant follicle present (≥ 19 mm), meaning the CL was functionally biosynthesizing estrogens and producing aromatase to stimulate this biosynthetic pathway. This spike in total estrogens might be interpreted as the preovulatory surge that accompanies estrus, yet it is derived from the CL rather than ovarian follicles. In this case, both the pregnant and unbred females exhibited elevated estrogen concentrations within their CL (Fig 2) providing further support that the unbred females were exhibiting a physiological pseudopregnancy, as elevated estrogen levels support fetal growth [46, 47]. The circulation of this hormone from the ovaries to the fetus would explain the lower concentrations found in the pregnant animals than the unbred females. Unbred female G150005 had 368.9 ng/g of total estrogens in her CL. This is slightly higher than the two pregnant females, yet interestingly the unbred female G110290 exhibited a concentration 3.7 times greater. The mean concentration for total estrogens of the pregnant females was nominally much smaller than that of unbred females. Female G150005 has been used in another study that analyzed progesterone and estradiol in bones, blubber and serum using liquid chromatography/tandem mass spectrometry (LC/MS/MS) [18]. The estradiol concentrations in the female from that study were low; 0.97 ng/g and 1.34 ng/ml in her blubber and serum respectively. Low estradiol in females in their study alluded the authors to conclude that females have low circulating estradiol concentration during the month of May. This is at odds with our results of elevated estrogens found within the CL of the unbred and pregnant animals hunted within the same time frame and location. The difference could be due to a lag in deposition of the hormone in blubber and circulating blood serum, or the less sensitive nature of LC/MS/MS compared to EIA for measuring concentrations of hormones, or that the former study measured solely estradiol, rather than total estrogens.

We could not determine differences in total estrogens for lactating versus non-lactating females. In humans, lactating women yield estradiol concentrations significantly lower than non-lactating women after 17 days or more postpartum [48]. Additionally, Ilcol et al. [49] found that lactating women 15–180 days postpartum had significantly lower levels of estradiol in their serum than those 1–3 days postpartum. One of the unbred female walruses in the present study was known to be lactating (G150005), and because both unbred females yielded similar hormone profiles, it is likely that both were lactating. Because female walruses typically nurse calves for 2–3 years [34], most sexually mature females (those that are selected by subsistence hunters on St. Lawrence Island) are typically producing milk. In our opportunistic study, no known individuals were not lactating, making us unable to determine the effect of lactation on estrogen concentrations.

Estrogen concentrations in the full-term pregnant female and the female in embryonic diapause were similar. It is thought that estradiol increases in concentration throughout pregnancy in pinnipeds [46]. In phocids, estradiol levels are flat during embryonic diapause until nidation, at which point plasma estradiol begins to increase and plateaus at parturition [46]. This was not the case in the two female walruses in the early and late phases of pregnancy, which had very similar levels of estrogen (Fig 2B). It may be that the concentrations in plasma do not immediately reflect ovarian concentrations, or that walruses do not follow this pattern witnessed in phocids.

### Progesterone

Interestingly, unbred females yielded the highest progesterone levels, even compared with the pregnant females. The unbred female used in Charapata et al. [18] (G150005) yielded among the greatest progesterone concentrations for all females in their sample; in her blubber and serum, progesterone levels were 110.87 ng/g and 18.04 ng/ml respectively. Recognizing that LC/MS/MS and EIAs are not necessarily comparable methods for measuring progesterone concentrations, these levels in the blubber and serum are still much lower than the concentration measured in her CL (1533.01 ng/g). Again, this indicates that the production site of this hormone maintains much greater concentrations than either the blood or blubber. Two of the nine postpartum females were considered outliers and had high levels of progesterone, similar to the concentrations in the pregnant animals (Fig 2A). These two postpartum females (S110008 and G110286) had some of the largest placental scar zone widths (both 13.5 cm) and heaviest tract weights (12.47 kg and 12.70 kg respectively), and they also had few follicles compared to other postpartum females. S110008 had 11 total follicles and G110286 had 7 follicles, whereas the mean for postpartum females was 28.2 follicles. While it is unknown whether or not the uterine lining produces progesterone, the large zone of placentation may have contributed to suppressing folliculogenesis and/or sustained progesterone production from the CL of the pregnancy. These factors indicate that these females were hunted very close to parturition, as Fay [34] found that placental scar widths of females that gave birth within 24 hours were 15 cm or greater. It appears that these CL were either still producing progesterone at the level of pregnancy, or were metabolizing it at a slower rate at the time these females were hunted than were the other postpartum walruses in the study. The CL of the two pregnant females had progesterone concentrations similar to the postpartum group, 99.4 ng/g and 77.5 ng/g, yet the two unbred females yielded progesterone concentrations 77 times greater than the postpartum females. It was expected that the greatest progesterone concentrations would be found in pregnant females as progesterone concentrations have been used as an accurate indicator of pregnancy in other marine mammals, though in different matrices such as blubber and serum [2, 3]. Of the two female walruses in the present study that were pregnant, G160045 was in embryonic diapause and her CL had 140.3 ng/g progesterone, whereas G160053 was full-term pregnant and had 58.5 ng/g progesterone. Both females had levels similar to postpartum individuals which ranged from 36.1–209.8 ng/g (mean 77.5 ng/g). In the female in embryonic diapause (G160045), this may indicate that the progesterone concentration remains low during embryonic diapause until nidation occurs. In that walrus, both of her reproductive hormone profiles mimicked that of the full-term pregnant female (Fig 2A & 2B) suggesting she was likely very close to implantation. An estrogen surge has been recorded to occur prior to nidation in several pinniped species including northern fur seals (*Callorhinus ursinus*) [50] and harbor seals [20], and may therefore be a signal of the end of embryonic diapause and re-activation of embryonic growth. Unfortunately, the uterus of the full-term pregnant animal was not collected, not allowing for an estimate of time from parturition using cervical dilation measurements.

### Pseudopregnancy

Pseudopregnancy has been defined as a state in which a non-gravid female shows clinical symptoms of pregnancy [51], during which the CL produces progesterone, often making pregnant and non-pregnant individuals endocrinologically indistinguishable [20, 21, 30]. Interestingly, the estrogen concentrations of unbred females were an order of magnitude greater than that of both pregnant females, making them distinguishably larger. Pseudopregnancy has been recorded in multiple pinniped species including Steller sea lions [21], harbor seals [20] and harp seals (*Phoca groenlandica*) [33]. The physiological duration of pseudopregnancy, in which the CL produces increased levels of progesterone, has been determined to be equivalent to that of embryonic diapause in other pinniped species [30, 33, 46, 52]. In the Pacific walrus, embryonic diapause is estimated to last 6 months [34], meaning pseudopregnancy symptoms could develop in individuals beginning in December (Fig 1). Assuming signs of gravidity appear after breeding commences in March, nonpregnant females may have elevated concentrations of progesterone April-September.

In the case of the full-term pregnant female walrus (G160053), the reduced level of progesterone production (in comparison to the unbred and pseudopregnant females) may indicate that at this phase in the pregnancy the placenta could be producing the required progesterone, however this has yet to be documented in pinnipeds. On the contrary, in ribbon seals (*Phoca fasciata*), spotted seals (*Phoca largha*) and Steller sea lions the placentae lack the enzymes capable of synthesizing progesterone, and it was determined that the CL is responsible for its production throughout the entire pregnancy [31, 53]. Studies on terrestrial carnivores including Japanese black bears (*Ursus thibetanus japonicus*) [32], and domestic dogs (*Canis lupus familiaris*) [16] have shown that the CL is the sole producer for progesterone even in late gestation, leading to the hypothesis that progesterone production in the placenta of carnivores may not be possible. There are several possible explanations as to why the two pregnant females exhibited progesterone profiles lower than their pseudopregnant conspecifics. In pregnant bitches there is a rapid decrease in progesterone 12–24 hours prepartum [16]. It could be that the full-term pregnant female was very close to parturition and her CL was already down-regulating progesterone production. It is also possible that pseudopregnant females generally have greater levels of CL progesterone than pregnant females because pregnant females are removing the available progesterone produced by the CL and it is being circulated to where it is needed, such as in the increasing and growing uterine, placenta and mammary glands. Concannon et al. [16] found that progesterone-releasing implants in nonpregnant bitches yielded concentrations 3 times greater than that of pregnant females with the same implants.

### Percent lipids

Percent lipids in ovarian tissues were analyzed to determine if they were a useful indicator of pregnancy. Percent lipids were greater in pregnant females (3.06%; *n* = 2) than postpartum females (1.60%; *n* = 9). Other relationships were investigated for percent lipids including harvest date, animal age, CL weight, CL dimension, reproductive tract weight, uterine horn width and placental zone width. No significant relationships were found when using linear regressions to compare these variables, with the exception of uterine horn width. There was a significant, positive relationship between the percent lipids found in CL and uterine horn width in the seven postpartum female walruses (Fig 3). Because the fetus develops in the uterine horn of this species [34] as in other pinnipeds, horn widths are greatest closest to parturition and diminish after birth with uterine involution. This supports the notion that percent lipids in CL may be a good indicator of time since parturition. Other studies that analyzed percent lipids in ovarian tissues of marine mammals were not found in the published literature, however percent lipids have been used to differentiate between reproductive classes of striped dolphins (*Stenella coeruleoalba*) in blubber [28] and their results showed that pregnant females had the largest mean percent lipids. Therefore, it could be expected that pregnant female walruses would have greater percent lipids in their ovaries than either postpartum or unbred females. When analyzing CL tissues, pregnant and unbred females had approximately twice the mean lipid content of postpartum females, though statistical significance was not able to be tested between groups, due to the small sample sizes available for this study. Again, the percent lipids in the CL of unbred females were indistinguishable from the pregnant females, providing further support for pseudopregnancy in the two unbred animals. It is worth noting that one of the unbred females had the largest percent lipid content in ovarian tissues of the four reproductive states, which would be characteristic of a capital breeder, where energy reserves are stored in preparation for and during pregnancy. Percent lipids are an indicator of energy gain and storage, which are important factors for females with or without offspring, and are key to the capital breeding reproductive strategy [54]. Postpartum walruses employ this capital breeding strategy, as little to no feeding is done in the first days or weeks following parturition [34]. It is likely that after pregnancy, lipids in the CL disperse outward into the surrounding ovarian tissue and are being mobilized for lactation. Again, small sample sizes precluded us from making a statistical comparison and these results should be interpreted with caution as there was large variability in the percent lipids of postpartum females. This study supports that analyzing CL tissues for lipid content may be a more useful technique than analyzing ovarian tissues for measuring time since parturition or determining reproductive status during this time of year. More samples are needed to determine if percent lipid content can differentiate among females in differing reproductive states.

### Follicular activity

Follicular activity was measured by comparing estrogen profiles within the follicles of large versus medium follicle sizes and in active and inactive ovaries, as well as the number of follicles per active and inactive ovary. While no significant differences could be found between groups, inactive ovaries tended to have slightly more follicles and slightly increased levels of estrogens (Fig 4). It was not surprising that inactive ovaries had more follicles than active ovaries as this is likely due to follicle stimulating hormone suppressing follicular activity in ovaries containing active CL. Active ovaries have been documented to have less follicles than inactive ovaries in other pinnipeds [50]. It was expected that larger follicles would yield greater estrogen concentrations, however, Fay [34] found that follicles were not mature until they reached a diameter of 19 mm, meaning all of the follicles in our sample were too small to be nearing ovulation and subsequent increased estrogen production. This is yet another indicator that the females in our sample were not exhibiting postpartum estrus, nor were they in a phase nearing estrus. Yet, it is plausible that walruses may experience a postpartum estrus later in the year as Fay also found that follicles in postpartum females reached their maximum size in July-August [34]. If a postpartum estrus does exist in late summer, it occurs 2–4 months following parturition, a longer timespan than has been reported in some other pinnipeds [46].

## Conclusions

In summary, the use of endocrine profiling to distinguish female walruses in various reproductive states is in its infancy but holds great potential. Specifically, profiling total estrogens of CL was useful in determining postpartum from non-postpartum females. This study presents the first documented physiological evidence for pseudopregnancy in walruses. CL tissues were a better indicator than ovarian tissues for determining reproductive status and percent lipids may also be a promising tool for determining reproductive status and time since parturition for postpartum females. Studies on walruses in human care have been carried out to determine if pregnancy and ovarian activity could be determined using vaginal fluid [17] and serum [55], though with limited success and applicability as these studies demonstrated that the reproductive hormones of animals in human care were not contiguous or synchronous with the breeding cycle of their free-ranging conspecifics. Future directions for these methods include estimating reproductive rates. Measuring progesterone in female cetacean tissues have been used to create pregnancy rate estimates [10, 12, 56, 57]. This technique could be extremely useful for walruses and should be validated in blubber and feces. Walruses are a particularly difficult species for obtaining reproductive rates because of their vast distribution, wary nature and harsh habitat to survey. Estimates for recent reproductive rates are lacking for this species [58, 59]. It has been nearly three decades since reproductive rates have been quantified and current management practices do not account for reproductive rates in models used to estimate population size [58, 59]. However, in order to determine pregnant from nonpregnant females during the potential pseudopregnancy phase in free-ranging walruses, we suggest 1) testing blubber samples and CL tissues when sampling in the spring, as blubber may acquire reproductive hormone signals more slowly than production sites and 2) sampling during fall and winter months, particularly from the end of September- end of November, when unbred females are less likely to be in a phase of pseudopregnancy (Fig 1). Further, sampling programs that collect female reproductive tracts from subsistence hunted, free-ranging animals already exist and could be utilized to assess reproductive rates of this hard-to-study species that is experiencing rapid environmental changes.

## Supporting information

S1 Table Concentrations of total estrogens in follicular fluid of female walruses. A indicates active ovaries and I indicates inactive ovaries. The concentrations of total estrogens were transformed using log_10_. Follicle sizes were determined as small (S; ≤ 1 mm), medium (M; 2–4 mm) and large (L > 4 mm); UNK indicates that follicle sizes were unknown and these samples were excluded in the size analysis of postpartum versus unbred females.
(DOCX)

S2 Table The number of follicles per ovary of female walruses. A indicates active ovaries and I indicates inactive ovaries. The number of follicles were transformed using log_10_.
(DOCX)

## References

[1] Atkinson S, Yoshioka M. Endocrinology of Reproduction. In: Miller D, editor. Reproductive Biology and Phylogeny of Cetacea: Whales, Porpoises and Dolphins. Boca Raton: CRC Press; 2007 10.1201/b11001.

[2] Amaral RS. Use of alternative matrices to monitor steroid hormones in aquatic mammals: a review. Aquat Mamm. 2010; 36:162–71.

[3] De Mello DMD, De Oliveira CA. Biological matrices for sampling free-ranging cetaceans and the implications of their use for reproductive endocrine monitoring. Mamm Rev. 2016; 46(2):1–15.

[4] Pérez S, García-López Á, De Stephanis R, Giménez J, García-Tiscar S, Verborgh P et al Use of blubber levels of progesterone to determine pregnancy in free-ranging live cetaceans. Mar Biol. 2011; 158:1677–80.

[5] Trego ML, Kellar NM, Danil K. Validation of blubber progesterone concentrations for pregnancy determination in three dolphin species and a porpoise. PLoS One. 2013; 8(7):1–9. 10.1371/journal.pone.0069709 PMC3728358 23936083

[6] Keller NM, Trego ML, Marks CI, Dizon AE. Determining pregnancy from blubber in three species of delphinids. Mar Mammal Sci. 2006; 22:1–16.

[7] Mansour AAH, McKay DW, Lien J, Orr JC, Banoub JH, ien N et al Determination of pregnancy status from blubber samples in minke whales (*Balaenoptera acutorostrata*). Mar Mammal Sci. 2002; 18:112–20.

[8] Birukawa N, Ando H, Goto M, Kanda N, Pastene LA, Nakatsuji H, et al Plasma and urine Levels of electrolytes, urea and steroid hormones involved in osmoregulation of cetaceans. Zoolog Sci. 2005; 22:1245–57. 16357473 10.2108/zsj.22.1245

[9] Kjeld M, Ólafsson Ö, Víkingsson GA, Sigurjónsson J. Sex hormones and reproductive status of the north Atlantic fin whales (*Balaenoptera physalus*) during the feeding season. Aquat Mamm. 2006; 32:75–84.

[10] Atkinson S, Gendron D, Branch TA, Mashburn KL, Melica V, Enriquez-Paredes LE, et al Pregnancy rate and biomarker validations from the blubber of eastern North Pacific blue whales. Mar Mammal Sci. 2020; 36:6–28.

[11] Valenzuela-Molina M, Atkinson S, Mashburn K, Gendron D, Brownell RL. Fecal steroid hormones reveal reproductive state in female blue whales sampled in the Gulf of California, Mexico. Gen Comp Endocrinol. 2018; 261:127–35. 29476760 10.1016/j.ygcen.2018.02.015

[12] Clark CT, Fleming AH, Calambokidis J, Kellar NM, Allen CD, Catelani KN, et al Heavy with child? Pregnancy status and stable isotope ratios as determined from biopsies of humpback whales. Conserv Physiol. 2016; 4:1–13. 27766149 10.1093/conphys/cow050 PMC5070529

[13] Kellar NM, Keliher J, Trego ML, Catelani KN, Hanns C, George JCC, et al Variation of bowhead whale progesterone concentrations across demographic groups and sample matrices. Endanger Species Res. 2013; 22:61–72.

[14] Connor RC, Richards AF, Smolker RA, Mann J. Patterns of female attractiveness in Indian Ocean bottlenose dolphins. Behaviour. 1996; 133:37–69.

[15] Kastelein RA, Vaughan N, Walton S, Wiepkema PR. Food intake and body measurements of Atlantic bottlenose dolphins (*Tursiops truncates*) in captivity. Mar Environ Res. 2002; 53:199–218. 11824828 10.1016/s0141-1136(01)00123-4

[16] Concannon PW, Castracane VD, Temple M, Montanez A. Endocrine control of ovarian function in dogs and other carnivores. Anim Reprod. 2009; 6:172–93.

[17] Kinoshita K, Kiwata M, Kuwano R, Sato N, Tanaka T, Nagata M, et al Temporal association of serum progesterone concentrations and vaginal cytology in walruses (*Odobenus rosmarus*). Theriogenology. 2012; 77(5):933–9. 22153266 10.1016/j.theriogenology.2011.09.024

[18] Charapata P, Horstmann L, Jannasch A, Misarti N. A novel method to measure steroid hormone concentrations in walrus bone from archaeological, historical, and modern time periods using liquid chromatography/tandem mass spectrometry. Rapid Commun Mass Spectrom 2018; 32:1999–2023. 30192037 10.1002/rcm.8272 PMC6282614

[19] Pietraszek J, Atkinson S. Concentrations of estrone sulfate and progesterone in plasma and saliva, vaginal cytology, and bioelectric impedance during the estrous cycle of the Hawaiian monk seal (*Monachus schauinslandi*). Mar Mammal Sci. 1994; 10(4):430–41.

[20] Reijnders PJH. Progesterone and oestradiol-17ß concentration profiles throughout the reproductive cycle in harbour seals (*Phoca vitulina*). J Reprod Fertil. 1990; 90:403–9. 2250239 10.1530/jrf.0.0900403

[21] Sattler R, Polasek L. Serum estradiol and progesterone profiles during estrus, pseudopregnancy, and active gestation in Steller sea lions. Zoo Biol. 2017 10.1002/zoo.21381 28901587

[22] Greig DJ, Mashburn KL, Rutishauser M, Gulland FMD, Williams TM, Atkinson S. Seasonal changes in circulating progesterone and estrogen concentrations in the California sea lion (*Zalophus californianus*). J Mammal. 2007; 88:67–72.

[23] Villegas-Amtmann S, Atkinson S, Costa DP. Low synchrony in the breeding cycle of galapagos sea lions revealed by seasonal progesterone concentrations. J Mammal. 2009; 90(5):1232–7.

[24] Rieger D, Loskutoff NM. Changes in the metabolism of glucose, pyruvate, glutamine and glycine during maturation of cattle oocytes *in vitro*. J Reprod Fertil 1994; 100:257–62. 8182598 10.1530/jrf.0.1000257

[25] Gardner DK, Lane M, Calderon I, Leeton J. Environment of the preimplantation human embryo in vivo: metabolite anlaysis of oviduct and uterine fluids and metabolism of cumulus cells. Fertil Steril. 1996; 65(2):349–53. 8566260 10.1016/s0015-0282(16)58097-2

[26] Krisher RL, Brad AM, Herrick JR, Sparman ML, Swain JE. A comparative analysis of metabolism and viability in porcine oocytes during *in vitro* maturation. Anim Reprod Sci. 2007; 98:72–96. 17110061 10.1016/j.anireprosci.2006.10.006

[27] Dunning KR, Cashman K, Russell DL, Thompson JG, Norman RJ, Robker RL. Beta-oxidation is essential for mouse oocyte developmental competence and early embryo development. Biol Reprod. 2010; 83:909–18. 20686180 10.1095/biolreprod.110.084145

[28] Gomez-Campos E, Borrell A AA. Assessment of nutritional condition indices across reproductive states in the striped dolphin (*Stenella coeruleoalba*). J Exp Mar Bio Ecol. 2011; 405(1):18–24.

[29] Hall G, Phillips TJ. Estrogen and skin: The effects of estrogen, menopause, and hormone replacement therapy on the skin. J Am Acad Dermatol. 2005; 53(4):555–68. 16198774 10.1016/j.jaad.2004.08.039

[30] Atkinson S. Reproductive biology of seals. Rev Reprod. 1997; 2:175–94. 9414481 10.1530/ror.0.0020175

[31] Ishinazaka T, Suzuki M, Yamamoto Y, Isono T, Harada N, Mason JI, et al Immunohistochemical localization of steroidogenic enzymes in the corpus luteum and the placenta of the ribbon seal (*Phoca fasciata*) and Steller sea lion (*Eumetopias jubatus*). J Vet Med Sci. 2001; 63(9):955–9. 11642282 10.1292/jvms.63.955

[32] Tsubota T, Taki S, Nakayama K, Mason JI, Kominami S, Harada N, et al Immunolocalization of steroidogenic enzymes in the corpus luteum and placenta of the Japanese black bear, *Ursus thibetanus japonicus*, during pregnancy. Reproduction. 2001; 121:587–94. 11277879 10.1530/rep.0.1210587

[33] Renouf D, Taylor R, Gales R. Pseudopregnancy harp seals (*Phoca groenlandica*). J Reprod Fertil. 1994; 101:31–6. 8064690 10.1530/jrf.0.1010031

[34] Fay FH. Ecology and Biology of the Pacific Walrus, *Odobenus rosmarus divergens* Illiger. North American Fauna. 1982; 74:1–279. Available from: http://www.fwspubs.org/doi/abs/10.3996/nafa.74.0001.

[35] Born EW. Reproduction in female Atlantic walruses (*Odobenus rosmarus rosmarus*) from north-west Greenland. J Zool. 2001; 255: 165–74.

[36] Perrin WF, Reilly SB. Reproductive parameters of dolphins and small whales of the family Deiphinidae. Rep Int Whal Comm. 1984; Special Issue (6):97–133. Available from: http://swfsc.noaa.gov/publications/CR/1984/8470.PDF.

[37] Tetsuka M, Asada M, Mogoe T, Fukui Y, Ishikawa H, Ohsumi S. The pattern of ovarian development in the prepubertal Antarctic minke whale (*Balaenoptera bonaerensis*). J Reprod Dev. 2004; 50(4):381–9. 15329469 10.1262/jrd.50.381

[38] Tarpley RJ, Hillmann DJ, George JC, Zeh JE, Suydam RS. Morphometric correlates of the ovary and ovulatory corpora in the bowhead whale, *Balaena mysticetus*. Anat Rec. 2016; 299:769–97. 10.1002/ar.23337 26917353

[39] Kesselring T, Viquerat S, Brehm R, Siebert U. Coming of age: Do female harbour porpoises (*Phocoena phocoena*) from the North Sea and Baltic Sea have sufficient time to reproduce in a human influenced environment? PLoS One. 2017; 13(6):1–14. 10.1371/journal.pone.0186951 PMC5650184 29053754

[40] Mashburn KL, Atkinson S. Evaluation of adrenal function in serum and feces of Steller sea lions (*Eumetopias jubatus*): influences of molt, gender, sample storage, and age on glucocorticoid metabolism. Gen Comp Endocrinol. 2004; 136:371–81. 15081837 10.1016/j.ygcen.2004.01.016

[41] Folch J, Lees M, Stanley GH. A simple method for total lipid extraction and purification. J Biol Chem. 1957; 226:497–509. 13428781

[42] Andrade JM, Estévez-Pérez MG. Statistical comparison of the slopes of two regression lines: A tutorial, Analytica Chimica Acta. 2014; 838:1–12. Available from: 10.1016/j.aca.2014.04.057. 25064237

[43] R Studio Team. 2019 RStudio: Integrated development for R. RStudio, Inc.: Boston.

[44] Hunt KE, Stimmelmayr R, George C, Hanns C, Suydam R, Brower H Jr, et al Baleen hormones: a novel tool for retrospective assessment of stress and reproduction in bowhead whales (*Balaena mysticetus*). Conserv Pysiology. 2014; 2:1–12. 10.1093/conphys/cou030 PMC4806734 27293651

[45] Champagne CD, Kellar NM, Trego ML, Delehanty B, Boonstra R, Wasser SK, et al Comprehensive endocrine response to acute stress in the bottlenose dolphin from serum, blubber, and feces. Gen Comp Endocrinol. 2018; 266:178–93. 29852162 10.1016/j.ygcen.2018.05.015

[46] Boyd IL. Environmental and physiological factors controlling the reproductive cycles of pinnipeds. Can J Zool. 1991; 69:1135–48.

[47] Mellish JE, Iverson SJ. Postpartum dynamics of reproductive hormones in gray and hooded seals. Mar Mammal Sci. 2005; 21:162–8.

[48] McNeilly AS. Effects of lactation on fertility. Br Med Bull. 1979; 2:151–4. 10.1093/oxfordjournals.bmb.a071562 387162

[49] Ilcol YO, Hizli ZB, Ozkan T. Leptin concentration in breast milk and its relationship to duration of lactation and hormonal status. Int Breastfeed J. 2006; 1:1–21. 10.1186/1746-4358-1-21 17109762 PMC1657001

[50] Shero MR, Bergfelt DR, Testa JW, Adams GP. Pairing ultrasonography with endocrinology to elucidate underlying mechanisms of successful pregnancy in the northern fur seal (*Callorhinus ursinus*). Gen Comp Endocrinol. 2018; 255:78–89. 29051074 10.1016/j.ygcen.2017.10.007

[51] Lueders I, Niemuller C, Steinmetz HW, Bouts T, Gray C, Knauf-Witzens T, et al Prolonged luteal lifespan and pseudopregnancy in Asian elephants (*Elephas maximus*). Anim Reprod Sci. 2018; 197:58–66. 30122269 10.1016/j.anireprosci.2018.08.008

[52] Gales NJ, Williamson P, Higgins LV, Blackberry MA, James I. Evidence for a prolonged postimplantation period in the Australian sea lion (*Neophoca cinerea*). J Reprod Fertil. 1997; 111:159–63. 9462281 10.1530/jrf.0.1110159

[53] Ishinazaka T, Suzuki M, Mizuno AW, Harada N, Mason JI, Ohtaishi N. Immunohistochemical localization of steroidogenic enzymes and prolactin receptors in the corpus luteum and placenta of spotted seals (*Phoca largha*) during late pregnancy. J Vet Med Sci. 2002; 64(4):329–33. 12014578 10.1292/jvms.64.329

[54] Houston AI, Stephens PA, Boyd IL, Harding KC, McNamara JM. Capital or income breeding? A theoretical model of female reproductive strategies. Behav Ecol. 2007; 18:241–250. Available from: 10.1093/beheco/arl080.

[55] Muraco HS, Coombs LD, Procter DG, Turek PJ, Muraco MJ. Use of human chorionic gonadotropin in a male Pacific walrus (*Odobenus rosmarus divergens*) to induce rut and achieve a pregnancy in a nulliparous female. J Androl. 2012; 33:789–97. 22207706 10.2164/jandrol.111.015032

[56] Kellar NM, Trego ML, Chivers SJ, Archer FI. Pregnancy patterns of pantropical spotted dolphins (*Stenella attenuata*) in the eastern tropical Pacific determined from hormonal analysis of blubber biopsies and correlations with the purse-seine tuna fishery. Mar Biol. 2013; 160:3113–24.

[57] Kellar NM, Trego ML, Chivers SJ, Archer FI, Perryman WL. From progesterone in biopsies to estimates of pregnancy rates: large scale reproductive patterns of two sympatric species of common dolphin, *Delphinus* spp. off California, USA and Baja, Mexico. Bull South Calif Acad Sci. 2014; 113(2):58–80.

[58] McCracken JG, Beatty WS, Garlich-Miller JL, Kissling ML, Snyder JA. Final species status assessment for the Pacific walrus (*Odobenus rosmarus divergens*). US Fish and Wildlife Service, Marine Mammal Management. 2017; 1:1–297.

[59] Larsen Tempel JT, Atkinson S. Pacific walrus (*Odobenus rosmarus divergens*) reproductive capacity changes in three time frames during 1975–2010. Polar Biol. 2020; 43:861–75.

