# Supplementary material for: Endocrine profiling of reproductive status and evidence of pseudopregnancy in the Pacific walrus (Odobenus rosmarus divergens)
Source: PLoS One. 2020 Sep 15;15(9):e0239218. doi: 10.1371/journal.pone.0239218 (PMC7491731; doi:10.1371/journal.pone.0239218)
Supplement: S1 Table — A indicates active ovaries and I indicates inactive ovaries. The concentrations of total estrogens were transformed using log10. Follicle sizes were determined as small (S; ≤ 1 mm), medium (M; 2–4 mm) and large (L > 4 mm); UNK indicates that follicle sizes were unknown and these samples were excluded in the size analysis of postpartum versus unbred females. (DOCX) [file pone.0239218.s001.docx]

| **Animal ID** | **Reproductive status** | **Activity** | **Total estrogens (ng/ml)** | **Log total estrogens (ng/g)** | **Size** |
| --- | --- | --- | --- | --- | --- |
| G110286 | postpartum | A | 4.5 | 0.66 | M |
| S110002 | postpartum | A | 2.4 | 0.37 | UNK |
| G110044 | postpartum | A | 1.3 | 0.13 | M |
| G110286 | postpartum | I | 5.3 | 0.72 | M |
| G110286 | postpartum | I | 4.0 | 0.60 | L |
| S110002 | postpartum | I | 9.0 | 0.95 | UNK |
| G110001 | postpartum | I | 42.2 | 1.63 | L |
| G110001 | postpartum | I | 33.6 | 1.53 | L |
| G110001 | postpartum | I | 7.6 | 0.88 | M |
| G110044 | postpartum | I | 2.4 | 0.38 | L |
| G110290 | unbred | A | 21.2 | 1.33 | M |
| G110290 | unbred | I | 28.5 | 1.46 | M |
