# Supplementary material for: Endocrine profiling of reproductive status and evidence of pseudopregnancy in the Pacific walrus (Odobenus rosmarus divergens)
Source: PLoS One. 2020 Sep 15;15(9):e0239218. doi: 10.1371/journal.pone.0239218 (PMC7491731; doi:10.1371/journal.pone.0239218)
Supplement: S2 Table — A indicates active ovaries and I indicates inactive ovaries. The number of follicles were transformed using log10. (DOCX) [file pone.0239218.s002.docx]

| **Animal ID** | **Activity** | **Number of follicles** | **Log number of follicles** |
| --- | --- | --- | --- |
| G110035 | A | 42 | 1.62 |
| G110044 | A | 12 | 1.08 |
| G110286 | A | 7 | 0.85 |
| G110290 | A | 32 | 1.51 |
| G110001 | A | 18 | 1.26 |
| S110002 | A | 21 | 1.32 |
| S110004 | A | 35 | 1.54 |
| S110008 | A | 11 | 1.04 |
| S110021 | A | 11 | 1.04 |
| G110035 | I | 76 | 1.88 |
| G110044 | I | 82 | 1.91 |
| G110286 | I | 45 | 1.65 |
| G110290 | I | 26 | 1.42 |
| G110001 | I | 0 | 0.00 |
| S110002 | I | 4 | 0.60 |
| S110008 | I | 18 | 1.26 |
| S110021 | I | 41 | 1.61 |
